# Complete Genome Sequence of Monkeypox Virus Strain MPXV-ROK-P1-2022 Isolated from the First Monkeypox Patient in the Republic of Korea

**DOI:** 10.1128/mra.00853-22

**Published:** 2022-10-17

**Authors:** Chi-Hwan Choi, Jin Sun No, Jin-Won Kim, Minji Lee, Hwachul Shin, Myung-Min Choi, Hwajung Yi, Gi-eun Rhie

**Affiliations:** a Division of High-risk Pathogens, Bureau of Infectious Diseases Diagnosis Control, Korea Disease Control and Prevention Agency, Cheongju, Republic of Korea; Queens College CUNY

## Abstract

We report the complete genome sequence of the monkeypox virus strain MPXV-ROK-P1-2022, isolated from the first patient diagnosed with monkeypox in the Republic of Korea in June 2022. The virus was fully sequenced on the Illumina MiSeq instrument, and the phylogenetic tree showed that the strain belongs to lineage B.1.1.

## ANNOUNCEMENT

**FIG 1:**
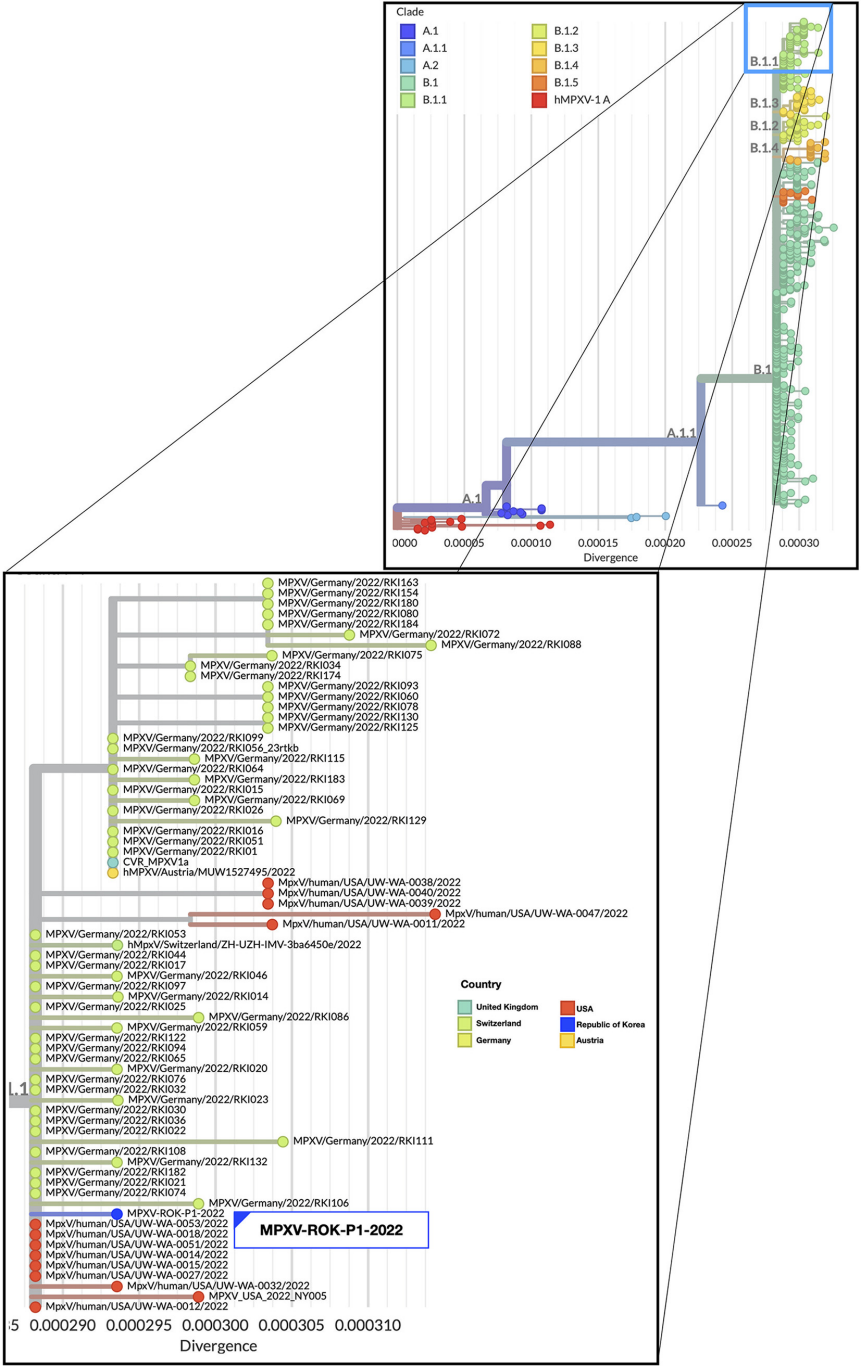
Phylogenetic tree of the monkeypox viruses using the Nextstrain analysis pipeline (https://github.com/nextstrain/monkeypox) (8). The tree shows that strain MPXV-ROK-P1-2022 belongs to lineage B.1.1.

Monkeypox is a rare and neglected zoonosis caused by infection with monkeypox virus, which is a member of the genus *Orthopoxvirus* in the family *Poxviridae*. According to an August 2022 report from the Johns Hopkins Center for Health Security (www.centerforhealthsecurity.org/resources/monkeypox), monkeypox has spread to countries where it is not endemic worldwide, with 32,000 confirmed cases and six deaths since the first case in May 2022. On 22 June 2022, the Korea Disease Control and Prevention Agency (KDCA) reported the first laboratory-confirmed case of monkeypox infection in the Republic of Korea (1). The monkeypox virus strain MPXV-ROK-P1-2022 was isolated from a swab of the patient’s skin lesion. To isolate the monkeypox virus from skin lesions, original swab eluates were incubated together with 2% fetal bovine serum (FBS)-Dulbecco’s modified Eagle medium (DMEM) and antibiotics (nystatin and penicillin-streptomycin) for 30 min at 4°C. After centrifugation, the supernatant was transferred to Vero E6 cell monolayers at 37°C. After 1 h incubation, cells were cultured in DMEM containing 10% FBS. After 72 h, cytopathic changes were observed, and viral DNA was extracted from the cell culture supernatant using the QIAamp DNA blood minikit (Qiagen GmbH, Hilden, Germany) in accordance with the manufacturer’s instructions. The MiSeq sequencing library was prepared according to the Illumina DNA preparation kit protocol (Illumina, San Diego, CA, USA). Sequencing was performed on the Illumina MiSeq instrument in 2 × 300-bp format using the MiSeq reagent kit v3 (Illumina) and in 2 × 150-bp format using the MiSeq reagent kit v2 (Illumina). Default parameters were used for all software unless otherwise specified. This yielded 49,636,800 (average length, 245 bp) and 57,173,318 (average length, 151 bp) reads, which were then filtered to a quality score of Q30 using Trimmomatic 0.39 (2) to remove adapters and low-quality sequences. Vero E6 host genome reads were filtered out using Bowtie2 (3). A total of 16,800,446 reads (v3, 5,642,934; v2, 11,157,512) were used for assembly with the resequencing method mapped to the reference genome sequence (GenBank accession number NC_063383) using CLC Genomics Workbench v.20.0 (Qiagen, Denmark). Final genome assembly was manually curated with contigs generated by *de novo* assembly with Unicycler v0.4.9 (4). Assembly quality and completeness were assessed using QUAST v5.0.2 (5) and CheckV v0.8.1 (6), respectively. The complete genome consisted of 197,200 bp (G+C content, 33%) (Table 1). The maximum-likelihood-based phylogenetic tree constructed with the general time-reversible (GTR) substitution model by IQ-TREE 2.1.2 (7) packaged into the Nextstrain analysis pipeline (8) shows that strain MPXV-ROK-P1-2022 belongs to lineage B.1.1 in clade IIb, which includes 70 strains from the United States (*n* = 14), Germany (*n* = 53), the United Kingdom (*n* = 1), Switzerland (*n* = 1), and Austria (*n* = 1) (Fig. 1).

**TABLE 1:** Assembly quality and completeness information for MPXV-ROK-P1-2022

Feature	Result for MPXV-ROK-P1-2022
GC content (%)^*a*^	33.0
Assembly length (bp)^*a*^	197,200
Length of longest contig (bp)^*a*^	197,200
No. of contigs^*a*^	1
*N*_50_ (bp)^*a*^	197,200
*L*_50_ ^*a*^	1
% mapped^*a*^	100
No. of total reads (left, right)^*a*^	592,704 (280,297, 280,297)
Avg coverage depth^*a*^	397
Genome fraction (%)^*a*^	100.0
CheckV quality^*b*^	Complete
Completeness^*b*^	100.0
Completeness method^*b*^	ITR^*c*^ (high confidence)

a QUAST.

b CheckV.

c Inverted terminal repeat.

In summary, we report the complete genome sequence of monkeypox virus strain MPXV-ROK-P1-2022, isolated from the first monkeypox patient in the Republic of Korea. The strain will be used as a reference strain for laboratory diagnosis of monkeypox at the KDCA.

This work was approved by the Institutional Review Board of KDCA (KDCA-IBC-2022-056).

### Data availability.

The complete genome sequence was deposited in DDBJ/ENA/GenBank under the accession number OP204857. The raw reads were deposited in the NCBI Sequence Read Archive under accession numbers SRX17096512, SRX17096513, SRX17096514, SRX17096515, SRX17096516, SRX17096517, SRX17096518, and SRX17096519.
